# Unsupervised Phonocardiogram Analysis With Distribution Density Based Variational Auto-Encoders

**DOI:** 10.3389/fmed.2021.655084

**Published:** 2021-08-05

**Authors:** Shengchen Li, Ke Tian

**Affiliations:** ^1^Department of Interlligent Science, Xi'an Jiaotong-Liverpool University, Suzhou, China; ^2^College of Electronic Engineering, Beijing University of Posts and Telecommunications, Beijing, China

**Keywords:** phonocardiogram analysis, auto-encoder, data density, unsupervised learning, abnormality detection

## Abstract

This paper proposes an unsupervised way for Phonocardiogram (PCG) analysis, which uses a revised auto encoder based on distribution density estimation in the latent space. Auto encoders especially Variational Auto-Encoders (VAEs) and its variant β−VAE are considered as one of the state-of-the-art methodologies for PCG analysis. VAE based models for PCG analysis assume that normal PCG signals can be represented by latent vectors that obey a normal Gaussian Model, which may not be necessary true in PCG analysis. This paper proposes two methods DBVAE and DBAE that are based on estimating the density of latent vectors in latent space to improve the performance of VAE based PCG analysis systems. Examining the system performance with PCG data from the a single domain and multiple domains, the proposed systems outperform the VAE based methods. The representation of normal PCG signals in the latent space is also investigated by calculating the kurtosis and skewness where DBAE introduces normal PCG representation following Gaussian-like models but DBVAE does not introduce normal PCG representation following Gaussian-like models.

## 1. Introduction

Phonocardiogram (PCG) analysis is a popular way for portable heart surveillance, which makes use of the heart sound to identify possible anomaly of heart statues. Existing PCG analysis methods use supervised methods which demands a labor expensive process of labeling. The paper proposes an unsupervised way of PCG analysis, which identifies abnormal PCG signals based on PCG analysis with normal signals only.

The main task of the proposed system is to characterize normal PCG signals in an unsupervised way and then identify abnormal PCG signals as outliers despite the existence of background noise and sound from other resources. In recent year, many attempts have been made to analyse PCG signals including the PhysioNet and CinC (Computing in Cardiology Challenge) data Challenge (1), which contains multiple sets of PCG data where both normal and abnormal PCG signals are presented and labeled.

With labels of normal and anomaly PCG signals, the PCG analysis can be considered as a classification problem. Classical machine learning techniques such as Support Vector Machine (SVM) (2), i-vector based dictionary learning method (3) and solutions based on Markov models (4) are used to solve the proposed problem besides deep learning algorithms (5, 6). However, as a supervised problem, PCG data collected needs to cover all types of PCG abnormality, which is labor expensive.

Inspired by the Anomalous sound detection (ASD) of Detection and Classification of Acoustic Scenes and Events (DCASE) data challenge 2020 (7, 8), the PCG analysis could also be considered as an unsupervised problem where only normal PCG signals are analyzed for the identification of anomaly PCG signals, which is considered as an outlier detection problem. This solution avoids PCG data collection problem as there is not need to collect all types of anomaly PCG signals for training.

The outlier detection of high-dimensional data is not a new research problem. Aggarwal and Yu (9) proposed to use sparse representation to find outliers. Pang et al. (10) using homophily couplings to identify outlier with noise. With the development of deep learning, Variational Automatic Encoder (VAE) (11) and a variant of VAE: β−VAE (12) are used for outlier detection in the PCG analysis, where the anomaly score of a PCG signal could be calculated by the features exacted from latent space of the VAE (13) or the reconstruction loss of β−VAE (14).

The PCG analysis based on VAE systems is based on an assumption that normal PCG signals can be represented by via latent vectors that obey a normal Gaussian distribution N(0,1). However, as normal PCG signals could be different from each other, the representation in latent space obeying a normal Gaussian distribution may not be the best feature representing PCG signals. For example, if the PCG collected from different sources, the PCG features could follow a Gaussian Mixture Model (GMM) due to different background noise and recording devices. In extreme cases, the resulting VAE may serve as a denoise VAE that converts anomaly PCG signals to normal PCG signals. As a result, this paper proposes two different ways to model normal PCG signals in a latent space.

The novelty of this paper is the use of sample density in latent space during the training process, which removes the assumption that normal PCG signals can be represented by latent vectors obeying a normal Gaussian distribution. At the same time, the KL divergence between latent vector distribution and normal Gaussian distribution is removed from the loss function, which potentially removes the assumption that the latent vectors must follow a normal Gaussian distribution.

Besides, the paper compares the system with and without the introduction of sampling process in the latent space during the training process. The proposed system with the sampling process in latent space follows the procedure that a VAE system is trained hence is named as Density based β−VAE system (DBVAE). The proposed system without the sampling process in the latent space likes a more traditional auto-encoder hence is named as Density based β−Auto-Encoder (DBAE) system. Both systems are compared with a β−VAE system, which is a more classical way for outlier identification.

The proposed method is tested with the Physio/CinC Heart Sound Dataset (1). There are six subsets of data collected, where each subset is collected in roughly the same way but from different places. This paper proposes two experiments to examine the performance of the proposed system. Firstly, the training data used is from the same subset. The resulting systems are evaluated by data from both the same subset and other subsets. Then data from different subsets are combined as the data used for training. The performance of the proposed systems are tested by the Receiver Operator Characteristic (ROC) test with Area Under Curve (AUC) values, which avoids the introduction of thresholds.

Theoretically speaking, the normal PCG representation in the latent space should follow a Gaussian-like model as there is a sampling process from Gaussian model during training. For the proposed DBAE, the resulting normal PCG representation in the latent space may not follow a Gaussian-like model due to the removal of sampling process from a Gaussian model. To examining the resulting normal PCG representation in the latent space, the kurtosis and skewness of the latent vectors are measured.

The paper is organized in the following way. Firstly, the proposed system is introduced. Then we present the results of the proposed experiments followed by the discussion to conclude this paper.

## 2. Methods

The proposed system is formed by three stages: pre-processing of the PCG signal, the training of the revised VAE system and the post-processing stage to produce the anomaly score, which is then evaluated by a Receiver Operator Characteristic (ROC) test for Area Under Curve (AUC) values.

### 2.1. Pre-processing

The Physio/CinC Heart Sound Dataset contains the audio of heart sound ranges from 5 to 120 s, effectively contains 6–13 cardiac cycles. For easier processing during the training process, a standardized 6-s length is used for all samples where longer samples are truncated and shorter samples are padded in a recurrent way.

As a common way to extract features, the Mel Spectrogram is calculated with the following configuration engaged: a window length of 1,024 with a hope length of 512. There are 14 Mel filters are used. As the sampling rate of the heart sound audio is 2 kHz, each frame engaged in the Mel Spectrogram lasts 0.51 s.

For data bias removal, the resulting coefficients in the Mel Spectrogram is standardized according to each row. Given a Mel Spectrogram ${\text{S}}_{M\times N}={\left[{\text{S}}_{1},{\text{S}}_{2},\ldots ,{\text{S}}_{M}\right]}^{T}$, the standardized row ${Ŝ}_{i}$ in a Mel Spectrogram can be written as

(1) $$\begin{array}{l} {Ŝ}_{i}=\frac{S_{i}-mean\left(S_{i}\right)}{std\left(S_{i}\right)}. \end{array}$$

The standardized Mel Spectrogram can be written as $Ŝ={\left[{Ŝ}_{1},{Ŝ}_{2},\ldots ,{Ŝ}_{M}\right]}^{T}$.

Each five frames of the standardized Mel Spectrogram then forms a super-frame, which is considered as a data sample in the training dataset. The starting frame of each super frame is selected in a rolling manner i.e., there are *L*−4 super-frames for a piece of audio with *L* frames. Each super-frame lasts about 3 s, which should contain at least one complete cardiac cycle.

### 2.2. Proposed Systems

The motivation of the proposed system is to relax the assumption that the use of VAE introduced in PCG analysis: there is a way to represent normal PCG signals whose representation in the latent space obeys a standardized Gaussian distribution. The assumption may cause two types of problems: (1) As VAE is commonly used as a de-noise system, the resulting VAE system could serve as a de-noise system for PCG signals which converts anomaly PCG signals to normal ones; (2) If the PCG signals are collected from multiple sources, the latent representation of PCG signals is unlikely to follow a single Gaussian model but a Gaussian Mixture model. As a result, there are two models proposed in this paper to solve the potential problems.

The first model is named “Density β−VAE” (DBVAE) that attempts to avoid the resulting latent representation of the normal PCG signals follows a normal Gaussian distribution if unnecessary. The DBVAE adopts a VAE system whose loss function is formed by the combination of reconstruction loss and the density of samples in the latent space. Adopted from the VAE framework, there is a re-sampling procedure from a Gaussian model in the latent space, which makes the representation of PCG signals in the latent space may potentially follow a Gaussian distribution. As a result, the DBVAE expects the PCG signals can be represented by latent vectors following a single-component Gaussian model.

**Figure 1:**
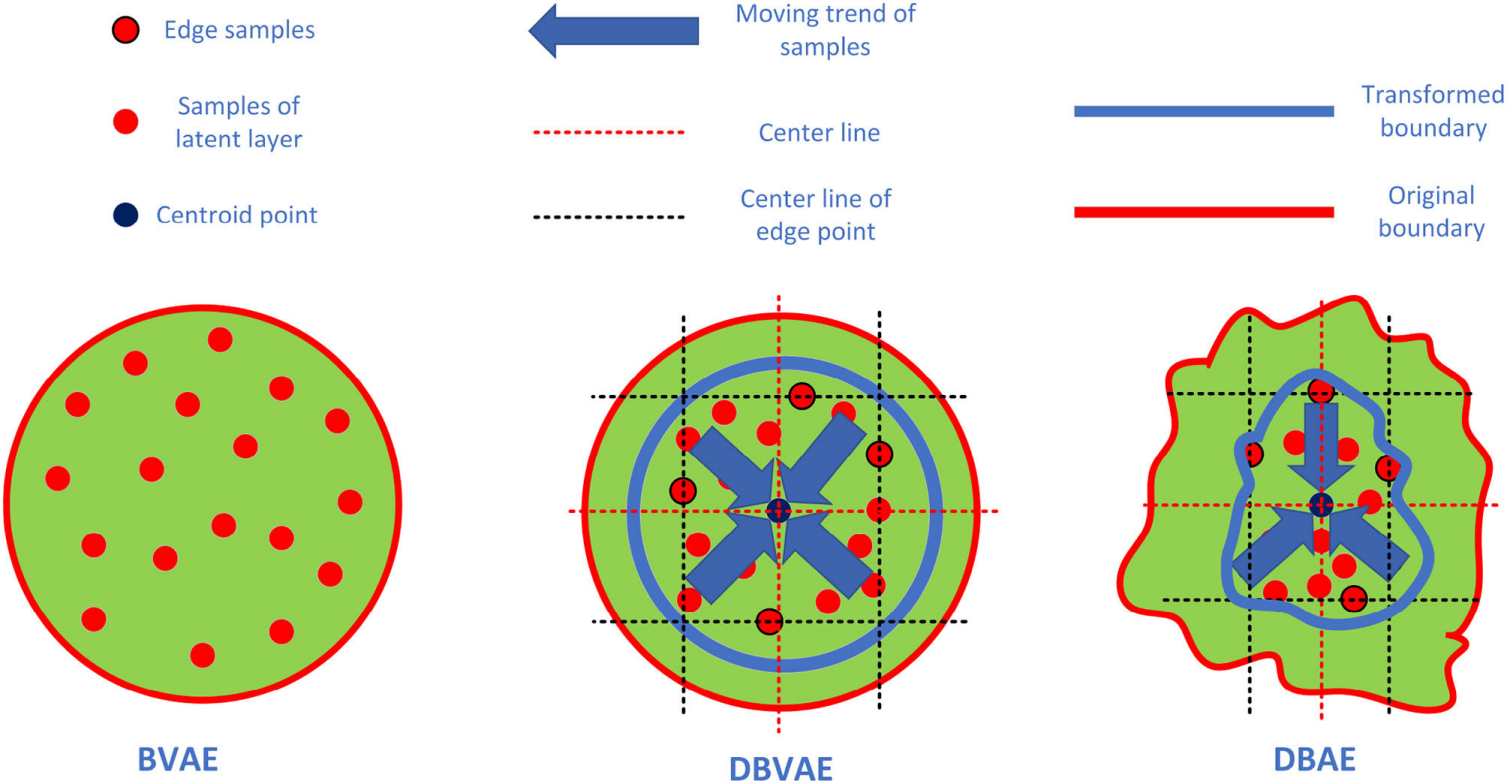
In the BVAE method, the latent space only ensures that the sample points roughly obey the standard normal distribution, but there is no specific requirement for the density of the sample points. In the DBVAE method, the latent space not only retains the characteristic that the sample points obey the standard normal, but also makes the samples more aggregated by increasing the sample density. The whole process is shown in the figure, taking two-dimensional plane as an example. Firstly, the edge sample is determined, and the center line of the edge point is made according to the center of the edge sample on each dimension. Secondly, the average of the center lines of the edge points is calculated to get the center line, and the intersection of the center lines of different dimensions is defined as the centroid point. Finally, all the sample points shrink to the centroid. In the method of DBAE, the distribution assumption of sample points is canceled and only the sample density is required. The samples shown in the figure are normal sample points during the training process.

If the training data collected is from multiple sources, latent representations for normal PCG signals resulted from DBVAE may not necessarily follow a single-component Gaussian model hence the “Density β−Auto Encoder” (DBAE) is introduced. By removing the re-sampling process in the latent space, the representation of normal PCG signals in latent space no longer follows a Gaussian distribution compulsory. The DBAE uses the same loss function with DBVAE, which pursues a high density distribution in the latent space. With the proposed loss function, DBAE could avoid overfitting in the latent space, which overcomes the problem of auto-encoders may have. Figure 1 gives a more intuitive explanation of the two methods.

The novel point of the proposed systems is to introduce a sample density based loss function term in the latent space. We now describe how sample density is estimated in the proposed systems.

In this paper, the sample density in latent space is defined as the average distance between each individual sample and the centroid point of the dataset. The centroid point of the dataset **C** = (*c*_1_, *c*_2_, …, *c*_*M*_) is formed by the centroid point of each dimension, where

(2) $$\begin{array}{l} c_{i}=\frac{max\left(z_{i1},z_{i2},\ldots ,z_{iN}\right)+min\left(z_{i1},z_{i2},\ldots ,z_{iN}\right)}{2}. \end{array}$$

The representation of all samples in the latent space is represented by **Z**_*M*×*N*_ whose *i*th dimension for the *j*th sample is represented as *z*_*ij*_. Using **Z**_*j*_ to represent the latent vector for sample *j*, The density measurement for all samples is then proposed as

(3) $$\begin{array}{l} D=\frac{1}{N}\overset{N}{\underset{j=1}{\sum }}||{\text{Z}}_{j}-\text{C}|{|}^{2}. \end{array}$$

Given $L_{r}$ to represent the reconstruction loss measure by Mean Squared Error (MSE), the overall loss functions for both DBVAE and DBAE are

(4) $$\begin{array}{l} L=L_{r}+\beta D. \end{array}$$

### 2.3. Post-processing

The anomaly score for a PCG signal is based on the reconstruction error of the proposed systems. For each super-frame (five consecutive frames) in Mel Spectrogram, the MSE between original Mel Spectrogram and the recovered Mel Spectrogram is considered as the anomaly score (*a*_*i*_) for this particular super-frame. The overall anomaly score (*a*) for a PCG signal with *N* frames is

(5) $$\begin{array}{l} a=\frac{1}{N-4}\overset{N-4}{\underset{i=1}{\sum }}a_{i}. \end{array}$$

## 3. Results

We firstly test the performance of the proposed systems with each single subset. Then we test the performance of the proposed system when how the subsets are combined. The baseline system selected is a β-VAE based system (14), which follows the extract experiment design in this paper.

There are six subset of data in the Physio/CinC dataset labeled as “a,” “b,” “c,” “d,” “e,” “f.” Given the fact that there are only a few samples in the subset “c,” the results for subset “c” is omitted when only a single subset is used as the data source for training. Besides the single subset tests, this paper also presents the experiments that use the combination of multiple subsets as the training data source. Specifically, the subsets with most data are tested (e.g., ‘a‘ & “e,” “e,” and “f”) and the case of all subsets used is also tests (subset “c” inclusive). In all cases, 90% normal PCG data is used for training and the remaining 10% normal PCG data and all anomaly PCG data are used for testing. In addition, in order to make the experiment more credible, this paper introduces an additional data set called Michigan (15). The experimental results are labeled “Michigan” with the same training proportion.

As discussed by Higgins et al. (12), in general β > 1 is necessary to achieve good disentanglement. However, as reported by Li et al. (14), a smaller β value may help the performance of PCG analysis. As a result, this paper sets the β values to wider range: 0.01, 0.1, 1, 10, and 100 to test how the value of β effects the performance of proposed systems.

As a summary, Table 1 shows the best and worst performed model for each type of candidate model with different settings of β values.

**Table 1:** Best and worst performed system for all tests in terms of AUC values (at the first line of each row).

**Best**	**a**	**b**	**d**	**e**	**f**	**ae**	**ef**	**ALL**	**Michigan**
BVAE (AUC)	0.825	0.559	0.691	0.923	0.846	0.822	0.899	0.786	0.966
*when* β:	*0.01*	*1.00*	*100*	*0.01*	*100*	*0.01*	*0.01*	*0.01*	*1.00*
DBVAE (AUC)	0.862	0.642	0.845	0.924	0.831	0.861	0.914	0.803	0.966
*when* β:	*0.1*	*0.01*	*0.1*	*0.01*	*10*	*0.01*	*0.01*	*0.01*	*1.00*
DBAE (AUC)	0.842	0.614	0.940	0.928	0.842	0.887	0.929	0.808	0.944
*when* β:	*0.1*	*1.00*	*0.1*	*0.1*	*10*	*0.1*	*100*	*1.00*	*0.01*
**Worst**	**a**	**b**	**d**	**e**	**f**	**ae**	**ef**	**ALL**	**Michigan**
BVAE (AUC)	0.798	0.551	0.583	0.881	0.801	0.765	0.836	0.644	0.725
*when* β:	*1.00*	*0.1*	*0.1*	*1.00*	*10*	*100*	*10*	*100*	*0.1*
DBVAE (AUC)	0.763	0.523	0.726	0.844	0.787	0.793	0.870	0.719	0.731
*when* β:	*100*	*0.1*	*100*	*100*	*1.00*	*100*	*100*	*10*	*0.1*
DBAE (AUC)	0.762	0.527	0.75	0.918	0.765	0.851	0.895	0.761	0.616
*when* β:	*10*	*100*	*0.01*	*1.00*	*100*	*10*	*10*	*100*	*1.00*

*The configuration of β is set as the second line of each row. The subsets used for training is labeled as the title of each column*.

From Table 2, the proposed DBAE and DBVAE systems generally outperform the BVAE system if the value of β is properly set. Specifically, when a single subset serves as the data source for training, the DBVAE has a comparable performance with DBAE in general whereas when multiple subsets are used as the data source for training, the DBAE in general outperforms the DBVAE and DBVAE is better than BVAE baseline.

**Table 2:** The ratio δ between the models with best β settings and the worst β setting in all experiments.

	**a**	**b**	**d**	**e**	**f**	**ae**	**ef**	**ALL**	**Michigan**
BVAE	0.034	0.018	0.185	0.048	0.051	0.074	0.076	0.220	0.332
DBVAE	0.131	0.217	0.164	0.095	0.056	0.085	0.050	0.118	0.321
DBAE	0.105	0.165	0.254	0.011	0.101	0.042	0.037	0.062	0.532

*A smaller number indicates less effects of β on system performance*.

Moreover, in the experiment presented, the results reveal that the effects of β differ from the candidate systems. Assuming the best performed β configuration is β_*b*_ and the worst performed β configuration is β_*w*_, Table 2 shows the value of $\delta =\frac{\beta_{b}}{\beta_{w}}-1$ for all experiments presented, which effectively measures how much performance be can gained by adjusting the value of β in extreme cases.

From results of δ, the effects of β value selection can be summarized as the following: (1) using multiple subsets generally reduce the effects on β value; (2) BVAE systems are more stable than DBVAE and DBAE when data from single subset is used; (3) DBAE improves the stability of system performance when multiple subsets are used for training.

## 4. Discussion

The proposed systems pursues different regulations on the distribution of latent vectors. To show how the PCG signals is presented in the latent space, the kurtosis and skewness are measured for the distribution of normal PCG signals. The definition of kurtosis and skewness is represented as follows.

Given a representation of PCG signal in the latent space [**Z**_*j*_ = (*z*_1*j*_, *z*_2*j*_, …, *z*_*Mj*_)] and the mean value of all latent vectors ($\bar{\text{Z}}$), the skewness (γ_1_) and kurtosis (γ_2_) of *N* samples in the latent space can be calculated as:

(6) $$\begin{array}{l} \gamma_{1}=\frac{\frac{1}{N}\sum_{i=1}^{N}{\left({\text{Z}}_{j}-\bar{\text{Z}}\right)}^{3}}{{\left(\frac{1}{N}\sum_{i=1}^{N}{\left({\text{Z}}_{j}-\bar{\text{Z}}\right)}^{2}\right)}^{3/2}} \end{array}$$

(7) $$\begin{array}{l} \gamma_{2}=\frac{\frac{1}{N}\sum_{i=1}^{N}{\left({\text{Z}}_{j}-\bar{\text{Z}}\right)}^{4}}{{\left(\frac{1}{N}\sum_{i=1}^{N}{\left({\text{Z}}_{j}-\bar{\text{Z}}\right)}^{2}\right)}^{2}}-3. \end{array}$$

Table 3 shows the average value and standard deviation of the skewness and kurtosis of the distribution in the latent space. For a normal Gaussian distribution, the skewness and kurtosis is expected to be 0. A larger kurtosis value indicates the distribution of latent vectors is more dense. A skewness value with higher absolute value is considered as more different with a normal Gaussian distribution.

**Table 3:** The average skewness and kurtosis for all resulting models in all experiments.

	**Skewness (γ_1_)**	**Kurtosis (γ_2_)**
N(0,1)	0	0
BVAE	0.115 (±0.093)	1.368 (±0.884)
DBVAE	2.674 (±8.128)	610.499 (±1669.592)
DBAE	−0.088 (±0.317)	3.369 (±4.413)

*The sign “±” represents then standard deviation of the data. N(0,1) represents normal Gaussian distribution*.

It is not surprising to find that BVAE systems produce a latent vector distribution that is similar with the normal Gaussian distribution. For DBAE, the resulting latent vectors in the latent space also follow a unbiased distribution with gentle variations on kurtosis in most cases, which suggests the resulting latent vectors follow a Gaussian-like model. Given the fact that for training data from multiple subsets should follow and mixture of models, it is interesting to find that the latent vectors as PCG normal signal representation follow a Gaussian-like model rather than a mixture of models. Moreover, it is surprising to find that the DBVAE results to heavily biased and high dense distribution despite a sampling process from Gaussian distribution, which suggests the resulting latent representation for DBVAE model is not following a Gaussian-like model. As a result, the normal PCG representation in the latent space needs further investigation in the future.

The motivation of proposing the DBVAE is to relax the assumption of the latent representation for normal PCG signals should follow a normal Gaussian distribution. The motivation of proposing DBAE is to relax the assumption of the latent representation for normal PCG signals should follow a Gaussian-like distribution. Both proposed system are expected to introduce an improvement of the system performance compared with VAE systems. Moreover, the DBAE is expected to outperform DBVAE when multiple subsets are used for training.

The final results confirm that both DBVAE and DBAE introduce an improvement on performance. DBAE introduces a small improvement compared with DBVAE when single subset is used as the source of training data. When multiple subsets are used for training, DBAE introduces a larger improvement compared with DBVAE. However, the investigation on the kurtosis and skewness of the distribution of PCG normal representation in latent space does not confirm the assumption this paper made where the DBVAE introduces a normal PCG representation in the latent space does not follow a Gaussian-like model but the DBAE introduces a normal PCG representation in the latent space that follows the a Gaussian-like model which are not expected.

As a quick conclusion, the introduction of density based auto-encoder systems, DBAE and DBVAE, improves the performance of PCG analysis however the latent representation of the proposed systems for normal PCG signals need investigation in the future for further improvements. The introduction of multiple subsets stabilizes the performance of the systems especially for DBAE, which reduces the efforts of tuning the value of β in the proposed systems.

## Data Availability Statement

Publicly available datasets were analyzed in this study. This data can be found here: the datasets analyzed for this study can be found in the PhysioNet at https://physionet.org/content/challenge-2016/1.0.0/.

## Author Contributions

SL composes the manuscript and designs the experiment proposed in the manuscript. KT implements the experiment for results with essential experiment design. All authors consider this piece of work as a full scale of collaboration.

## Conflict of Interest

The authors declare that the research was conducted in the absence of any commercial or financial relationships that could be construed as a potential conflict of interest.

## Publisher's Note

All claims expressed in this article are solely those of the authors and do not necessarily represent those of their affiliated organizations, or those of the publisher, the editors and the reviewers. Any product that may be evaluated in this article, or claim that may be made by its manufacturer, is not guaranteed or endorsed by the publisher.
